# Can Environmental Manipulation Help Suppress Cancer? Non‐Linear Competition Among Tumor Cells in Periodically Changing Conditions

**DOI:** 10.1002/advs.202000340

**Published:** 2020-07-01

**Authors:** S. G. Babajanyan, Eugene V. Koonin, Kang Hao Cheong

**Affiliations:** ^1^ Science and Math Cluster Singapore University of Technology and Design S487372 Singapore; ^2^ National Center for Biotechnology Information National Library of Medicine National Institutes of Health Bethesda MD 20894 USA

**Keywords:** cancer suppression, cancer treatment, evolutionary dynamics, game theory, non‐linear dynamics, population dynamics, predatory–prey

## Abstract

It has been shown that the tumor population growth dynamics in a periodically varying environment can drastically differ from the one in a fixed environment. Thus, the environment of a tumor can potentially be manipulated to suppress cancer progression. Diverse evolutionary processes play vital roles in cancer progression and accordingly, understanding the interplay between these processes is essential in optimizing the treatment strategy. Somatic evolution and genetic instability result in intra‐tumor cell heterogeneity. Various models have been developed to analyze the interactions between different types of tumor cells. Here, models of density‐dependent interaction between different types of tumor cells under fast periodical environmental changes are examined. It is illustrated that tumor population densities, which vary on a slow time scale, are affected by fast environmental variations. Finally, the intriguing density‐dependent interactions in metastatic castration‐resistant prostate cancer (mCRPC) in which the different types of tumor cells are defined with respect to the production of and dependence on testosterone are discussed.

## Graphical abstract

The models of density‐dependent interaction between different types of tumor cells under fast periodical environmental changes are examined. Non‐linear dynamical effects in the tumor growth process are discovered. These dynamical effects can be manipulated for controlling the tumor growth process. The density‐dependent interactions in metastatic castrate‐resistant prostate cancer are also discussed.

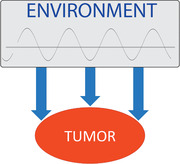

## 1 Introduction

Darwinian adaptive evolution is one of the key processes in tumor progression.^[^ ^1^, ^2^, ^3^, ^4^, ^5^, ^6^, ^7^, ^8^, ^9^, ^10^, ^11^, ^12^, ^13^, ^14^ ^]^ Diversification and adaptation of tumor cells is a reason for cancer treatment failures and multi‐drug resistance of tumor cells.^[^ ^14^, ^15^, ^16^, ^17^, ^18^, ^19^ ^]^ For optimization of treatment strategies, it is widely accepted that tumor evolution processes must be taken into consideration.^[^ ^14^, ^18^, ^20^, ^21^, ^22^, ^23^, ^24^, ^25^, ^26^, ^27^ ^]^ These strategies focus on the suppression of tumor proliferation by taking advantage of evolutionary factors.

Any tumor consists of different types of cells^[^ ^28^, ^29^ ^]^ that interact with one another and with normal cells. Various mathematical models have been suggested for describing the interaction between different types of tumor cells and between normal and tumor cells.^[^ ^5^, ^6^, ^7^, ^8^, ^9^, ^10^, ^11^, ^12^, ^27^, ^30^, ^31^, ^32^, ^33^ ^]^

The vital role of the microenvironment in tumor initiation and proliferation processes is well‐recognized.^[^ ^2^, ^3^, ^10^, ^11^, ^14^, ^15^, ^34^, ^35^, ^36^, ^37^ ^]^ The microenvironment can promote or suppress tumor proliferation.^[^ ^10^, ^11^ ^]^ Changes in the microenvironment can occur as a result of interaction between tumor and stromal cells and because of biotic (nutrients supply) and abiotic (drug application) variation.^[^ ^10^, ^11^, ^24^, ^35^ ^]^ Because the environment affects the tumor evolution, these changes represent potential targets for antitumor treatment.^[^ ^10^, ^31^, ^32^, ^37^, ^38^ ^]^ Adaptive treatment strategies strive to exploit these interactions to control the tumor growth, instead of killing as many tumor cells as possible.

Both frequency‐dependent selection (where the dynamics of heritable traits does not depend on the total population size) and density‐dependent population growth dynamics (where the interaction between different types within the population depends on the population size) of cells subject to environmental changes can drastically differ from those in a fixed environment.^[^ ^39^, ^40^, ^41^, ^42^, ^43^, ^44^, ^45^, ^46^, ^47^, ^48^, ^49^, ^50^ ^]^ A changing environment can induce polymorphic states that do not exist in fixed environment,^[^ ^40^, ^43^, ^45^, ^47^, ^51^, ^52^ ^]^ thus changing the evolutionary dynamics^[^ ^53^, ^54^ ^]^ of the population.

In a changing environment, the density‐dependent population growth dynamics also differs from that in a fixed environment. The changing environment can vary both spatially and temporally. The analysis presented here pertains to a time‐variable, periodically changing environment. Periodical changes affecting the tumor cells can be associated with biotic (blood circulation, nutrients supply) or abiotic (periodical drug usage) variations and/or mechanical stress; periodical variations are defined with respect to the characteristic lifetime of the population. It has been shown that periodical environmental changes allow stable coexistence of two competing species in cases when such coexistence is impossible in a fixed environment.^[^ ^41^, ^43^, ^45^, ^47^, ^48^ ^]^ Here, we focus on fast environmental changes, that is, the case when the environment goes through multiple oscillations during the characteristic time of the population growth. These oscillations are considered as periodical variations of the characteristics of tumor population growth. It is assumed that environmental changes affect the competition between different cell types. However, these changes occur on a fast time‐scale, so that the averaged competition coefficients (over the period of environmental variations) of different types of tumor cells do not vary during the population growth time. It is also possible to assume that the competing cell types do not change in the course of the population growth, that is, we examine the population dynamics without mutations, so that the change of the population size depends only on density‐dependent competition (no migration). We observe that, due to the fast environmental changes, a new mechanism of competition between different types can arise in the population dynamics. The dynamics of the density‐dependent population growth depend on the total size of the population, and thus, arising new mechanisms can also alter the growth dynamics of the entire tumor. This information can be crucial for tumor growth suppression.^[^ ^21^ ^]^ These observations may hold promise for a novel approach for suppression of tumor growth for cancer treatment.

The proposed model is applicable to tumors that contain different types of cells competing for resources. As an illustration of the effects caused by environmental changes, we analyze the tumor population growth of the metastatic castration‐resistant prostate cancer (mCRPC) in a changing environment.The mCRPC contains three distinct cell types that differ with respect to the production of and dependence on testosterone,^[^ ^33^, ^55^ ^]^ and we elucidate the density‐dependent interactions between those cell types.

The interactions between different types of tumor cells define the evolutionary outcome of the tumor population growth. The growing success of a cell type is defined by its reproduction (reproduction coefficients) and competition (coefficients matrix) rates. The reproduction rate corresponds to the population growth in the absence of other types. The competition matrix defines the competition between different cell types, that is, how the presence of one type will affect the dynamics of the other type. In mCRPC, the worst outcomes occur when the competition matrix supports the existence of only *T* ^−^ cell types (these are the type of tumor cells that are independent of testosterone) or a high density of this type in the population as an evolutionary outcome, because the *T* ^−^ type cells are not vulnerable under ADT and abiraterone therapies. In this work, we focus on these two cases, namely the occurrence of *T* ^−^ type cells only as an outcome of the population growth dynamics in a fixed environment and the occurrence of high density of the *T* ^−^ type. For these cases, the effect of environmental changes on the tumor population growth is explored by mathematical and computer simulations, coupled with analytic derivations.

## 2 Results

### 2.1 Population Model

We first assume that there are *n* types of competing species (cell types). The density‐dependent population dynamics of these species is described by the Lotka–Volterra system of equations. We are interested in both the complete population size and the composition of different species in the population. As such, we use the Lotka–Volterra system written in terms of the population densities and the total number of individuals in the population.^[^ ^56^ ^]^

(1) $$\begin{array}{cc} \frac{dp_{i}}{dt} & =p_{i}\left(f_{i}-\sum_{j}p_{j}f_{j}\right),\;\sum_{i}p_{i}=1 \end{array}$$

(2) $$\begin{array}{cc} f_{i} & \equiv r_{i}-N\sum_{j}a_{ij}p_{j}\\ \frac{dN}{dt} & =N\sum_{j}p_{j}f_{j} \end{array}$$

where *N* is the total population size, $p_{i}=\frac{N_{i}}{N}$ is the density (fraction) of type *i*, *r*_*i*_ is the reproduction rate for type *i*, *a*_*ij*_ > 0 is the competing coefficient between species {*ij*}, and *f*_*i*_ is the fitness of the population *i*. Positive *a*_*ij*_ means that the growth of a type *i* is negatively affected by type *j*; in general, *a*_*ij*_ ≠ *a*_*ji*_. In prey–predator models, some of these coefficients can be negative, that is, the presence of prey promotes the growth of predators. It can be seen that Equation (1) is similar to the replicator equation, which is known from the frequency‐dependent selection in evolutionary dynamics.^[^ ^51^, ^52^, ^57^ ^]^ However, in contrast to the replicator dynamics where the fitness does not depend on the population size, under density‐dependent population growth, the fitness of the population is a decreasing function of the population size. In this model, at equilibrium, the fitness of each population type cancels out as seen from (1) and (2), if all types are present in the equilibrium state *p*_*i*_ > 0, i=1,…,n. In the stable state, the population size does not vary (2), hence the mean fitness is zero. From this, it follows that the fitness of each type is zero in the stable state, if each type is initially present in the population. From the definition of *p*_*i*_ and (1), it follows that $\sum_{i}p_{i}=1,\;\sum_{i}\frac{dp_{i}}{dt}=0$. Hence, the possible compositions of the population are located in the simplex Σ^*n*^ for any given population size *N*, where each vertex of the simplex represents the population state consisting of only one type. For each point in this simplex, there is an equilibrium population size.^[^ ^56^ ^]^

Environmental changes are incorporated as fast and periodical changes in the competition coefficients. We assume that the reproduction coefficients do not vary with time. The competition coefficients consist of a constant ${\bar{a}}_{ij}$ and a time oscillating term ${\tilde{a}}_{ij}\left(\tau \right)$.

(3) $$\begin{array}{ccc} a_{ij}\left(\tau \right) & = & {\bar{a}}_{ij}+{\tilde{a}}_{ij}\left(\tau \right) \end{array}$$

where τ = ω*t* is the fast time. The constant part in (3) describes the interaction between species without any environmental changes (either biotic or abiotic). These coefficients define the population dynamics outcome described by the system of Equations (1) and (2). The oscillating term describes the effect of the biotic and abiotic variations on the competition. It is reasonable to assume that the time‐average of the term for each period of environmental changes is zero.

(4) $$\begin{array}{ccc} \bar{{\tilde{a}}_{ij}} & \equiv & \int_{0}^{2\pi }{\tilde{a}}_{ij}\left(\tau \right)\frac{d\tau }{2\pi }=0 \end{array}$$

After each period of environmental change, the competition coefficients will have the same values as before in the variations.

The population dynamics in the fast time‐scale describe the instantaneous impact of variation in the environment on the population densities. By contrast, the slow time‐scale dynamics describe the evolutionary outcome. These two types of dynamics are in a feedback relation. The fast variations in the population densities depend on the composition of the population in the slow time (i.e., over longer time periods), and conversely, the fast variations indirectly affect the evolution of population densities in the slow time. The periodical variation of the environment induces a new mechanism in the tumor population growth dynamics. In the presence of periodical variations of the environment, competition between different tumor cell types changes: essentially, the success of a type of cells is defined not only by the competition with other types separately, but also by the competition with two or more other types simultaneously. These new effects are due to the overlap between time‐dependent competition rates, and those can potentially be exploited as a controlling mechanism for tumor population growth.

### 2.2 Tumor Population Growth under Environmental Changes

Here, we address tumor population growth under environmental changes for two cases, using mCRPC as an example. The mCRPC contains three distinct cell types. The *T* ^+^ type cells require exogenous androgen and rely on the testosterone produced by the human body. Androgen deprivation therapy (ADT) that stops the normal production of testosterone^[^ ^58^ ^]^ is used against this type of cells. However, resistance to the ADT builds up over time. One of the causes for the resistance is the increased expression of the CYP17A enzyme^[^ ^17^, ^55^ ^]^ that is essential for testosterone production. As a result, the cancer cells start to produce testosterone. The second type of tumor cells are the testosterone‐producing cells *T* ^*p*^. These cells occur as a result of the up‐regulation of the CYP17A enzyme. Against this type, the abiraterone therapy is used, which blocks the production of CYP17A.^[^ ^33^, ^55^ ^]^ The third type are *T* ^−^ cells, which do not produce testosterone and do not rely on it. It has been shown that incorporating the evolutionary dynamics into the treatment procedure leads to improved results.^[^ ^33^, ^55^ ^]^ In some cases, it is also possible to suppress the growth of *T* ^−^ cells.^[^ ^55^ ^]^ These cases are defined by the competition coefficients, which can vary for different patients.

There are two distinct types of tumor growth dynamics. Under the first type, the only stable rest point of the tumor population growth dynamics in a fixed environment is the vertex of *T* ^−^ type; that is, the tumor consists entirely of *T* ^−^ cells. In the second case, the population dynamics in a fixed environment also has only one rest point, but it is not in a vertex of the simplex, that is, more than one type is present in the stable state. The stability of the vertices in the fixed environment is defined from the system ((1), (2)). The vertex *i*, *i* = 1, 2, 3 (where 1,2,3 refer to the cases for which the tumor consists of only *T* ^+^, *T* ^*p*^, and *T* ^−^ cells, respectively) will be a stable rest point of the system ((1), (2)) in the fixed environment if

(5) $$\begin{array}{c} r_{i}{\bar{a}}_{ki}>r_{k}{\bar{a}}_{ii},\;i,k=1,2,3,\;i\neq k \end{array}$$

If condition (5) is not satisfied for all vertices, then the stable rest point of the system is in the interior of the simplex. Thus, in this case, there is more than one type of tumor cells in the equilibrium state. In the former case, the environmental changes can induce a new stable rest point in the interior of the simplex for the slow‐varying population densities. In the latter case, where there can be more than one type of tumor cells represented in the stable state, environmental variations can cause changes in the stationary densities of tumor cells. Hence, the number of stable rest points remains the same. For illustrative purposes, we use data drawn from the cancer research literature.^[^ ^55^ ^]^ The coefficients equal to {*r*_1_, *r*_2_, *r*_3_} = {0.278, 0.355, 0.665} are reproduction rates determined by cell lines, and represent the upper bound for reproduction rates.^[^ ^55^ ^]^ Thus, it is reasonable to use these values to illustrate possible non‐trivial dynamical effects.

For the given reproduction coefficients, the stable equilibrium state of the population dynamics is defined by the competition coefficients. The ordering of the possible competition coefficients is defined by two rules^[^ ^55^ ^]^: first, in the absence of exogenous testosterone, *T* ^+^ tumor type cells are the least competitive type; the second rule is that the competitive effect of the *T* ^−^ type against the *T* ^*p*^ type is stronger than against the *T* ^+^ type. These two rules define six inequalities between the competition coefficients *a*_31_ > *a*_21_, *a*_32_ > *a*_12_, *a*_13_ > *a*_23_, *a*_23_ > *a*_21_, *a*_13_ > *a*_12_, and *a*_32_ > *a*_31_. The possible scenarios of tumor population growth in terms of these competing coefficients in the fixed environment, and for the frequency‐dependent selection mechanism in the tumor are discussed in refs. [7, 32, 33, 55]. With respect to the population growth dynamics, these six inequalities should be considered under the condition (5), to define the possible stable state in the fixed environment. Hence, we will use two different competition coefficient matrices for the two scenarios discussed above.

#### 2.2.1 Coexistence of Different Cell Types Due to Environmental Variation

We now investigate the scenario under which the dominance of *T* ^−^ cells is inevitable. This means that the only stable rest point of the population dynamics in the fixed environment is the vertex in which all cells are of type *T* ^−^. The competition coefficient matrix which satisfies (5) for the above conditions has the following form:

**Table:** 

	*T* ^+^	*T* ^*p*^	*T* ^−^
*T* ^+^	1/*K*	0.7/*K*	0.8/*K*
*T* ^*p*^	0.4/*K*	1/*K*	0.5/*K*
*T* ^−^	0.6/*K*	0.9/*K*	1/*K*

where *K* is a large number. The appearance of *K* is due to the fact that in a stable state, the total population size has to be sufficiently large. Otherwise, in the stable state, the total population size is equal to *r*_3_ found from (2). Hereafter we assume *K* = 10 000. The dynamical properties of population growth does not depend on the exact value of *K*.

**Figure 1:**
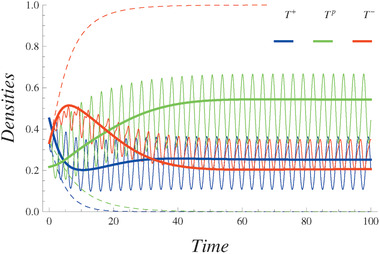
Densities of the tumor types–*T* ^+^(blue), *T* ^*p*^(green), and *T* ^−^(red). For ω = 2, κ_1_ = κ_2_ = 3.35*10^−8^. The coefficients κ_*i*_ describe the advantage of type *i* in the competition with other types due to the environmental changes. Initial conditions are as follows $p_{1}\left(0\right)=p_{2}\left(0\right)=p_{3}\left(0\right)=\frac{1}{3}$, *N*(0) = 3000. The initial conditions of the slow time varying densities ${\bar{p}}_{1}\left(0\right)=0.447,{\bar{p}}_{2}\left(0\right)=0.219,{\bar{p}}_{3}\left(0\right)=0.333$. Dashed lines are the densities of different types of tumor cells in the fixed environment found from ((1), (2)). Solid lines represent the slow‐varying densities. These quantities are obtained from (11) and (12). Oscillating curves represent the densities of different types directly found from the system (1), (2), and (3).

For the above parameters, the population growth dynamics in fixed and periodically changing environments are illustrated in **Figure** 1. Only *T* ^−^ cells are present in the stable state.

For the environmental changes, we assume ω = 2 and the competition coefficients ${\tilde{a}}_{ij}$ are harmonic functions such that κ_1_ = κ_2_ = 3.35 × 10^−8^. The coefficients κ_*i*_ describe the advantage of type *i* in the competition with other types due to the environmental changes. These quantities arise due to the overlaps between periodically varying parts of the competition coefficients. The oscillating curves represent the solution of the dynamical system (1) and (2) using competition coefficients (3). In contrast to the case of the fixed environment, in the periodically changing environment, each cell type is represented in the equilibrium state (see the solid lines showing the time‐averaged densities in Figure 1).

The appearance of the tumor cell types *T* ^+^ and *T* ^*p*^ in the equilibrium state is due to the non‐linear competition terms in the population growth dynamics (11) and (12). The tumor cell types *T* ^+^ and *T* ^*p*^ obtain an advantage in the competition due to environmental variations. These advantages are proportional to the coefficient κ = κ_1_ = κ_2_. Meanwhile, from the definition of the non‐linear competition coefficients (12), it follows that the tumor type *T* ^−^ is disadvantaged by the environmental variations. It can be seen that the behavior of the slow time varying densities coincides with the non‐averaged densities, determined numerically without the separation of the fast and slow quantities. Indeed, this result clearly validates our approach of separating the slow‐ and fast‐varying quantities. The behavior of these densities depends on the frequency of the environmental variations ω. This approach is valid for frequencies ω > 1; otherwise all quantities that are of order $O(\frac{1}{\omega^{2}})$ cannot be ignored (see Section 4 for details).

The non‐linear terms will not change the stability of the vertices. Thus, for the cases where the stable rest point of the population growth dynamics (in fixed environment) is in a vertex, that vertex will also be a stable rest point in the case of the varying environment. Accordingly, by changing the initial state of the population dynamics in a varying environment, the tumor growth dynamics might end up in the *T* ^−^ vertex in the current context.

**Figure 2:**
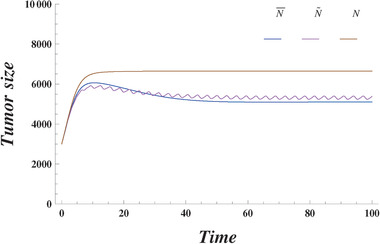
Tumor population size. For ω = 2, κ_1_ = κ_2_ = 3.35*10^−8^. Initial conditions are as follows $p_{1}\left(0\right)=p_{2}\left(0\right)=p_{3}\left(0\right)=\frac{1}{3}$, *N*(0) = 3000. The initial conditions of the slow time varying densities ${\bar{p}}_{1}\left(0\right)=0.447,{\bar{p}}_{2}\left(0\right)=0.219,{\bar{p}}_{3}\left(0\right)=0.333$. The averaged population size defined from (12) is represented by the blue line. The magenta line represents the total population size directly found from ((1), (2)) and (3). The brown line represents the population size in the fixed environment (κ_1_ = κ_2_ = 0).

The dynamics of the total population size is shown in **Figure** 2. The initial conditions and the environmental variations are the same as above. In the stable state, the total population size in the fixed environment is larger than that in the varying environment. This is again due to the fact that the environmental variations create a new stable state in the population dynamics. Indeed, if the initial state is changed such that the dynamics converges to the vertex of *T* ^−^ cells, then the total population size will be the same for the fixed and varying environments.

**Figure 3:**
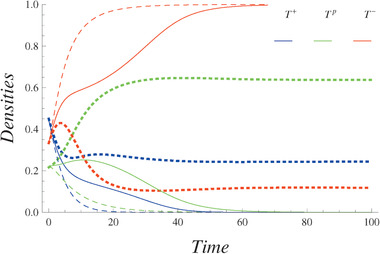
Density dynamics of tumor cells in various changing environment. The frequency of the environmental variations are the same ω = 2. Dashed lines represent competition between different types of tumor cells in the fixed environment κ = κ_1_ = κ_2_ = 0. Solid lines illustrate the competition for  κ = 2.95*10^−8^. In this case, the environmental variation does not generate a new stable state for the population dynamics. However, the environmental variations increase the time taken for the population dynamics to converge to the unique stable state. The dotted lines represent population dynamics for  κ = 3.35*10^−8^. The initial conditions are the same as in Figure 1.

The appearance of the new stable rest point in the population dynamics depends on the values of the non‐linear competition coefficient (**Figure** 3). The dotted lines represent the dynamics of the population densities in the fixed environment κ = κ_1_ = κ_2_ = 0. Dotted lines represent the population growth dynamics in the varying environment for the case when the environmental variations induce new stable state of the population dynamics. If the values of non‐linear competition coefficients κ_*i*_ are not sufficiently large, then the population growth dynamics end up in the same state as in the fixed environment (solid lines in the figures, for  κ = 2.95*10^−8^).^[^ ^45^, ^47^ ^]^ However, the environmental variations can increase the time taken for the population dynamics to reach the stable state. For any given time, the density of *T* ^−^ tumor type is higher in the fixed environment than in the varying environment.

In the given varying environment, the appearance of the new stable rest point also depends on the initial population size. Under the initial conditions discussed, initial conditions for tumor population $p_{1}\left(0\right)=p_{2}\left(0\right)=p_{3}\left(0\right)=\frac{1}{3}$, but for a small total population size *N*(0) = 300, the new stable state does not appear in the population dynamics. This effect is due to the dependence of the non‐linear terms on the square of the population size (11). The non‐linear terms are small for the small populations. To create a new stable state for the small population, higher coefficients of environmental variations κ are needed. Thus, the composition and the population size of a tumor are equally important for controlling the tumor population dynamics.^[^ ^21^ ^]^

#### 2.2.2 Controlling a Pre‐Existing Heterogenous Tumor Cell Population

Here, we consider the effect of a varying environment on the population dynamics for which the stable state in a fixed environment includes more than one type of tumor cells. The competition coefficients have to satisfy the six inequalities discussed above, whereas the condition (5) might not be satisfied for any vertex.

The competition coefficient matrix is as follows. Under these parameters, the condition (5) does not hold for any vertex. Thus, none of the vertices is a stable state of the population growth dynamics in the fixed environment. However, the six inequalities for the competition coefficients are satisfied.

**Table:** 

	*T* ^+^	*T* ^*p*^	*T* ^−^
*T* ^+^	1/*K*	0.6/*K*	0.7/*K*
*T* ^*p*^	0.4/*K*	1/*K*	0.5/*K*
*T* ^−^	0.8/*K*	0.9/*K*	1/*K*

**Figure 4:**
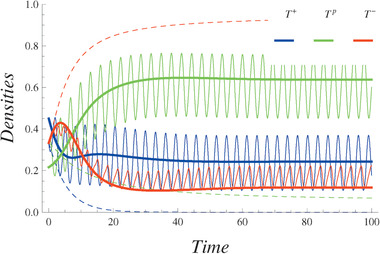
Densities of the tumor types—*T* ^+^(blue), *T* ^*p*^(green), *T* ^−^(red). For ω = 2, κ_1_ = κ_2_ = 3.35*10^−8^. The initial conditions are the same as in Figure 1. Dashed lines represent tumor types dynamics in the fixed environment found from ((1), (2)). Full lines represent the slow‐varying densities obtained from ((11), (12)). Oscillating curves represent the densities of different types directly found from the system (1), (2), and (3).

The population growth dynamics of tumor types in the fixed and varying environments are shown in **Figure** 4. In Figure 4, the initial state of the system is the same as the condition in Figure 1.

In this case, the *T* ^+^ type does not occur in the equilibrium state in the fixed environment. The density of the *T* ^−^ type is much greater than the density of the *T* ^*p*^ type. The equilibrium state is on the edge of the simplex connecting the vertices *T* ^−^ and *T* ^*p*^.

Environmental variations can change the densities of the different types of tumor cells in the already established coexistence. In contrast to the case of a fixed environment, the majority of the tumor cells in the new stable state in the varying environment are of type *T* ^*p*^. Conversely, the *T* ^−^ cells are now the minority in the tumor cell population.

In this context, the stable state of the population growth dynamics in the fixed environment is also a stable state of the population growth dynamics in the varying environment. This is the case because the stable state of the population growth dynamics in the fixed environment is on the edge of the simplex so that the non‐linear terms cancel out (11). In general, when the rest point is in the interior of the simplex, the stable state of the population growth dynamics in the fixed environment will not be a rest point of the dynamics in the varying environment, as follows from (11) and (12).

In the previous case, the total population size in the new stable state is smaller than that in the stable state in the fixed environment. Indeed, the closer the stable state is getting to the vertex of the *T* ^−^ cells in the simplex, the larger the total population size becomes. This follows from (1) and (2), and from the ordering of the reproduction and competition coefficients.

The new stable state generation is possible for relatively small values of non‐linear competition coefficients κ_*i*_, *i* = 1, 2 compared to the previous case.

## 3 Discussion

Here, we have investigated the tumor population growth dynamics in a periodically varying environment assuming that the environmental changes are fast compared to the tumor growth dynamics. In particular, we examined the competition dynamics between different types of tumor cells in constant and periodically varying environments. These periodical changes can be associated with biotic (blood circulation, nutrients supply) or abiotic (periodical drug usage) variations and/or mechanical stresses. The environmental variations substantially affect the competitiveness of different tumor types but do not alter the time averages of the competition coefficients over a period of environmental variations, which are equal to those in a fixed environment. The environment‐dependent variations in the competition coefficients induce the fast oscillations of the tumor population densities, whereas the slow‐varying component of the densities represents the population growth.

Although many researchers have hypothesized that environmental variations can drastically change the tumor population growth dynamics, our present work provides a comprehensive model and analysis of the phenomenon.^[^ ^2^, ^3^, ^10^, ^11^, ^14^, ^15^, ^31^, ^32^, ^34^, ^35^, ^36^, ^37^, ^38^ ^]^ When the competition coefficients vary in the same way, that is, there is no overlap between the varying parts of these coefficients, the population growth dynamics (for slow‐varying densities) does not respond to the environmental variations; that is, in this case, the varying environment is effectively equivalent to a fixed environment. This finding has to be taken into account in cancer therapy approaches that involve changing the environment of a tumor by designing these changes such that the competition coefficient values overlap.

Of special interest are the environment‐induced variations in the competing coefficients when a zero‐sum condition is imposed on the competing coefficients. Under the zero‐sum condition, the tumor population dynamics obtains new non‐linear competition terms. Now the competitive success of the given type depends not only on its competition with the other types, but also on the competition between those other types. Thus, a type that is successful in the competition in a fixed environment, can lose in the varying environment. This effect has been noted in several papers,^[^ ^41^, ^45^, ^47^, ^48^ ^]^ but these results are of limited applicability because the analytic derivations are effectively intractable or address specific cases only. Crucially, we linked tumor growth dynamics to environmental variations. Under the zero‐sum condition (i.e., if a tumor cell type *i* gets an advantage, in terms of competition coefficients, over type *j*, the latter type loses the same amount in the competition with the former type), the total population size is indirectly affected by the environmental variations, that is, there are no additional terms in the population size dynamics.

More specifically, we examine the competition between *T* ^+^, *T* ^*p*^, and *T* ^−^ cell types in the metastatic castration‐resistant prostate cancer (mCRPC). These three cell types fundamentally differ with respect to the population of and dependence on testosterone. We describe the competition between these cell types under zero‐sum condition and focus on two types of dynamics: a) when the dominance of *T* ^−^ cells is inevitable, such that, in a fixed environment, only this type of cell is represented in the equilibrium state and b) high percentage of the tumor cells are of type *T* ^−^ in the equilibrium state. The tumor population dynamics is determined by the reproduction and competition coefficients. The densities of each type of tumor cells in the evolutionary outcome of tumor population dynamics are defined by these coefficients. In the real world, these coefficients can vary between patients. Although our results can be generalized for each possible ordering of the competition coefficients, we only discuss the worst case scenario here, namely, the dominance of *T* ^−^ cells that are not susceptible to hormone therapies.

- For the first case, it has been shown that the environmental variations can induce a new stable state of population growth dynamics. In this state, all the competing types are represented. Meanwhile, the equilibrium state of the population dynamics in the fixed environment remains unchanged. Thus, the population growth will be defined by the initial state of the tumor population or by the basin of attraction of stable states. The total population size of the tumor cells is smaller in the new environment‐induced stable state than it is in the stable state in the fixed environment. If the population growth dynamics converges to the original stable state (which is the only stable state in the fixed environment that includes only *T* ^−^ cells) in the presence of environmental variations, then the population size will also be the same as that in the absence of variations. The appearance of the new stable state depends on the values of non‐linear competition coefficients such that for small values (weak competition), the new stable state may be inaccessible. Although environmental variations can slow down the growth of *T* ^−^ cells, the dominance of these cells will still be inevitable.
- In the second case, there is more than one type of tumor cell in the stable state of the tumor population dynamics. The environmental variations can change the densities of the tumor cell types. Here, we discuss the case in which two type of tumor cells are represented in the stable state, *T* ^−^ cells with a high density and *T* ^*p*^ cells with a low density. In this case, the original stable state is also a equilibrium state for the population dynamics in the varying environment. However, for the case of an equilibrium state where all cell types are present, the latter observation no longer holds. In the example discussed above, the total population size in the new stable state is also smaller than that in the equilibrium state in a fixed environment.

In conclusion, we show here that environmental variations can drastically change the tumor population growth dynamics. These environmental variations induce a new competition mechanism in the tumor population. These non‐linear competition mechanisms can prevent the dominance of a type of tumor cell that inevitably dominates in the fixed environment. Thus, these variations can be exploited for controlling the tumor population growth such that the tumor population becomes vulnerable to milder treatment methods.

## 4 Experimental Section

We first represent the population densities *p*_*i*_(*t*) by its slow ${\bar{p}}_{i}\left(t\right)$ and fast‐varying parts $\varepsilon_{i}\left(\bar{p},N,\tau \right)$ using the Kapitza method.^[^ ^59^ ^]^

(6) $$\begin{array}{ccc} p_{i}\left(t\right) & = & {\bar{p}}_{i}\left(t\right)+\varepsilon_{i}\left(\bar{p}\left(t\right),N\left(t\right),\tau \right) \end{array}$$

Let ${\hat{a}}_{ij}$ be the primitive of ${\tilde{a}}_{ij}$, that is, $\partial_{\tau }{\hat{a}}_{ij}={\tilde{a}}_{ij}$ and $\bar{{\hat{a}}_{ij}}=0$. Note that ${\hat{a}}_{ij}$ is also a periodic function with the same period. The slow‐varying part ${\bar{p}}_{i}\left(t\right)$ represents the density dynamics of the different types of tumor cells in slow times. The fast‐varying part $\varepsilon_{i}\left(\bar{p}\left(t\right),N\left(t\right),\tau \right)$ represents the changes in the population due to the environmental variations. The latter is smaller than the slowly varying part ${\bar{p}}_{i}\left(t\right)$, oscillates in the fast time and averages to zero:

(7) $$\begin{array}{ccc} {\bar{\varepsilon }}_{i}\left(\bar{p}\left(t\right),N\left(t\right)\right) & \equiv & \int_{0}^{2\pi }\varepsilon_{i}\left(\bar{p}\left(t\right),N\left(t\right),\tau \right)\frac{d\tau }{2\pi }=0 \end{array}$$

The population growth dynamics in the slow time *t* is derived using the system of Equations (1) and (2) with (3), (6), and (7), expanding the right hand‐side of (1) by $\varepsilon_{i}\left(\bar{p}\left(t\right),N\left(t\right),\tau \right)$ and keeping the quantities which are of order $O(\frac{1}{\omega })$, finally averaging by fast time τ (see Supporting Information).

(8) $$\begin{array}{cc} \frac{d{\bar{p}}_{i}}{dt} & ={\bar{p}}_{i}\left(F_{i}-\sum_{j}{\bar{p}}_{j}F_{j}\right),\;\sum_{i}p_{i}=1 \end{array}$$

(9) $$\begin{array}{cc} F_{i} & \equiv r_{i}-N\left(\sum_{j}{\bar{a}}_{ij}p_{j}-\right.\\ & \left.-N\left(\sum_{k,l}b_{ikl}{\bar{p}}_{k}{\bar{p}}_{l}+\sum_{m,n,l}c_{ikmn}{\bar{p}}_{k}{\bar{p}}_{m}{\bar{p}}_{n}\right)\right)\\ \frac{dN}{dt} & =N\sum_{j}{\bar{p}}_{j}F_{j} \end{array}$$

The fast environmental changes contribute to the population dynamics by incorporating the non‐linear terms, concerning the competing coefficients in the growth rates of the population densities. These coefficients have the following form:

(10) $$\begin{array}{c} b_{ikl}=\frac{1}{\omega }\bar{{\tilde{a}}_{ik}{\hat{a}}_{kl}},\;c_{ikmn}=\frac{1}{2\omega }\bar{{\hat{a}}_{ik}\left({\tilde{a}}_{mn}+{\tilde{a}}_{nm}\right)} \end{array}$$

The effective dynamics of the population growth in the slow time differs from the known density‐dependent growth of the population. Indeed, the growth rates of the population densities do not linearly depend on the population size *N*. The periodical variations of the environment effectively change the nature of the population growth dynamics. Note that the initial state for the averaged densities in (8) and (9) differs from the initial state of the non‐averaged densities in (1) and (2) (see Supporting Information). The latter effect is known as the “initial slip” in dynamical systems.^[^ ^60^ ^]^

From (8), the density of the given type depends not only on the competition of different pairs, but also on the competition of the given type against the total population in fast‐time scales. The intensities of these interactions are described by the averaged coefficients (10) and by the total population size.

The non‐linear terms in population growth rates cancel out if all the coefficients vary in the same phase ${\tilde{a}}_{ij}=\alpha_{ij}g\left(t\right)$ for all types–α_*ij*_, amplitudes of varying coefficients, or if only one coefficient varies during the time. Thus, if the biotic or abiotic variations of the tumor environment change the competition between different types in the same way, then the varying environment can be considered to be a fixed one. The environmental changes induce a non‐trivial contribution to the population growth dynamics if at least two competition coefficients vary in different phases. The necessity of varying phase overlapping for non‐trivial effects of the population growth in the periodical changing environment has been recognized.^[^ ^45^, ^47^, ^48^ ^]^

The non‐linear terms also cancel out in the limit of ω → ∞. Thus, the environment‐induced effects in the population growth dynamics will be non‐trivial if the environment does not vary too fast. Thus, in the presence of the environmental changes (either biotic or abiotic), the population growth dynamics can drastically differ from the dynamics under fixed environment.

Here, we will study the population growth dynamics comprising three types under the periodical environmental changes. The case of three types in the population corresponds to the different types of tumor cells in metastatic prostate cancer—*T* ^+^, *T* ^*p*^, and *T* ^−^ cells. The densities of the different types of tumors are denoted by *T* ^+^ → *p*_1_, *Tp* → *p*_2_, *T* ^−^ → *p*_3_.

It is assumed that the self‐limitation of different types are not affected by the environmental changes ${\tilde{a}}_{ii}=0,\;i=1,2,3$. For the rest of the competition coefficients, a zero‐sum condition ${\tilde{a}}_{ij}=-{\tilde{a}}_{ji}$ is made. The latter condition means that if the type *i* takes an advantage over type *j*–due to the environmental variations–then the latter type suffers by the same amount in the competition with the former type.

Under the above conditions and from (8) and (9), the population growth dynamics for three types of competing species takes the following form:

(11) $$\begin{array}{cc} \frac{d{\bar{p}}_{i}}{dt} & ={\bar{p}}_{i}\left({\bar{f}}_{i}-\sum_{j}{\bar{p}}_{j}{\bar{f}}_{j}\right)+\kappa_{i}N^{2}{\bar{p}}_{1}{\bar{p}}_{2}{\bar{p}}_{3} \end{array}$$

(12) $$\begin{array}{cc} {\bar{f}}_{i} & \equiv r_{i}-N\sum_{j}{\bar{a}}_{ij}{\bar{p}}_{j}\\ \frac{dN}{dt} & =N\sum_{j}{\bar{p}}_{j}{\bar{f}}_{j} \end{array}$$

where *i* = 1, 2, 3 and

$$\begin{array}{cc} \kappa_{1}=b_{123} & +b_{132},\;\kappa_{2}=b_{213}+b_{231}\\ & \kappa_{1}+\kappa_{2}+\kappa_{3}=0 \end{array}$$

The sum of non‐linear terms in (11) cancels out because it is needed for the conservation of normalization $\sum_{i=1}^{3}{\bar{p}}_{i}=1$.

Equation (11) reveals that the non‐linear terms do not change the stability of the vertexes in the simplex. This is because the non‐linear terms cancel out at each vertex. Thus, if a vertex is a stable rest point in the fixed environment described by (1) and (2), then it will remain as a stable rest point under the environment‐induced dynamics and with zero‐sum condition. From (12), it follows that under the zero‐sum condition the environment‐induced non‐linear terms are absent from the total population growth dynamics. This is due to the fact that the non‐linear averaged terms in (9) nullify under zero‐sum condition. Thus, the environmental changes indirectly affect the total population growth dynamics, that is, only by the averaged population densities.

The coefficients κ_*i*_ in (11) describe the advantage taken by the type *i* in competition with other types due to the environmental changes, for example, for *T* ^+^ type, the coefficient κ_1_ includes two terms *b*_123_ and *b*_132_. Each of these terms includes the competition coefficient of type *T* ^+^ with the other two types. Using the zero‐sum condition, the coefficient κ_1_ can be written as $\kappa_{1}=\frac{1}{\omega }\bar{\left({\tilde{a}}_{12}-{\tilde{a}}_{13}\right){\hat{a}}_{23}}$. It is seen that the possible advantage of type *T* ^+^ against other types depends on both the competition of that type with other types and the competition between the other two types. Note that the values of the non‐linear coefficients κ_*i*_ do not depend on the fixed competition coefficients ${\bar{a}}_{ij}$ because the population growth dynamics in slow times is obtained from (1) and (2) under the assumption of ${\bar{p}}_{i}>>\varepsilon_{i}$.

## Conflict of Interest

The authors declare no conflict of interest.

## Supporting information

Supporting Information

## References

[1] P. C. Nowell , Science 1976, 194, 23.959840

[2] D. Hanahan , R. A. Weinberg , Cell 2011, 144, 646. 21376230 10.1016/j.cell.2011.02.013

[3] R. A. Gatenby , R. J. Gillies , Nat. Rev. Cancer 2008, 8, 1450021. 10.1038/nrc2255 18059462

[4] P. M. Altrock , L. L. Liu , F. Michor , Nat. Rev. Cancer 2015, 15, 730. 26597528 10.1038/nrc4029

[5] N. Beerenwinkel , R. F. Schwarz , M. Gerstung , F. Markowetz , Syst. Biol. 2014, 64, e1. 25293804 10.1093/sysbio/syu081 PMC4265145

[6] M. Greaves , C. C. Maley , Nature 2012, 481, 306. 22258609 10.1038/nature10762 PMC3367003

[7] J. M. Pacheco , F. C. Santos , D. Dingli , Interface Focus 2014, 4, 20140019. 25097748 10.1098/rsfs.2014.0019 PMC4071510

[8] S. Hummert , K. Bohl , D. Basanta , A. Deutsch , S. Werner , G. Theißen , A. Schroeter , S. Schuster , Mol. BioSyst. 2014, 10, 3044. 25270362 10.1039/c3mb70602h

[9] D. W. Strand , O. E. Franco , D. Basanta , A. R. A. Anderson , S. W. Hayward , Curr. Mol. Med. 2010, 10, 95. 20205682 10.2174/156652410791065363 PMC4195241

[10] L. M. Merlo , J. W. Pepper , B. J. Reid , C. C. Maley , Nat. Rev. Cancer 2006, 6, 924. 17109012 10.1038/nrc2013

[11] K. Polyak , I. Haviv , I. G. Campbell , Trends Genet. 2009, 25, 30. 19054589 10.1016/j.tig.2008.10.012

[12] M. Archetti , K. J. Pienta , Nat. Rev. Cancer 2018, 19, 110. 10.1038/s41568-018-0083-7 PMC8557269 30470829

[13] M. C. Lloyd , J. J. Cunningham , M. M. Bui , R. J. Gillies , J. S. Brown , R. A. Gatenby , Mol. Cancer Ther. 2016, 76, 3136. 10.1158/0008-5472.CAN-15-2962 PMC5384728 27009166

[14] C. A. Aktipis , R. M. Nesse , Evol. Appl. 2013, 6, 144. 23396885 10.1111/eva.12034 PMC3567479

[15] L. A. Garraway , P. A. Jänne , Cancer Discovery 2012, 2, 214. 22585993 10.1158/2159-8290.CD-12-0012

[16] M. Gerlinger , C. Swanton , Br. J. Cancer 2010, 103, 1139. 20877357 10.1038/sj.bjc.6605912 PMC2967073

[17] R. B. Montgomery , E. A. Mostaghel , R. Vessella , D. L. Hess , T. F. Kalhorn , C. S. Higano , L. D. True , P. S. Nelson , Cancer Res. 2008, 68, 4447. 18519708 10.1158/0008-5472.CAN-08-0249 PMC2536685

[18] J. J. Cunningham , R. A. Gatenby , J. S. Brown , Mol. Pharmaceutics 2011, 8, 2094. 10.1021/mp2002279 PMC3250072 21815657

[19] R. A. Gatenby , Nature 2009, 459, 508. 19478766 10.1038/459508a

[20] K. J. Pienta , N. McGregor , R. Axelrod , D. E. Axelrod , Translational Oncology 2008, 1, 158. 19043526 10.1593/tlo.08178 PMC2582164

[21] K. S. Korolev , J. B. Xavier , J. Gore , Nat. Rev. Cancer 2014, 14, 371. 24739582 10.1038/nrc3712 PMC13213539

[22] P. A. Orlando , R. A. Gatenby , J. S. Brown , Br. J. Cancer 2012, 9, 065007. 10.1088/1478-3975/9/6/065007 PMC3653600 23197192

[23] P. M. Enriquez‐Navas , J. W. Wojtkowiak , R. A. Gatenby , Cancer Res. 2015, 75, 4675. 26527288 10.1158/0008-5472.CAN-15-1337 PMC4693617

[24] R. A. Gatenby , T. L. Vincent , Mol. Cancer Ther. 2003, 2, 919. 14555711

[25] D. Basanta , R. A. Gatenby , A. R. Anderson , Mol. Pharmaceutics 2012, 9, 914. 10.1021/mp200458e PMC3325107 22369188

[26] J. A. Gallaher , P. M. Enriquez‐Navas , K. A. Luddy , R. A. Gatenby , A. R. Anderson , Cancer Res. 2018, 78, 2127. 29382708 10.1158/0008-5472.CAN-17-2649 PMC5899666

[27] A. Kaznatcheev , R. Vander Velde , J. G. Scott , D. Basanta , Br. J. Cancer 2017, 116, 785. 28183139 10.1038/bjc.2017.5 PMC5355932

[28] J. D. Nagy , Mathematical Biosciences and Engineering 2005, 2, 381. 20369929 10.3934/mbe.2005.2.381

[29] M. Gerlinger , A. J. Rowan , S. Horswell , J. Larkin , D. Endesfelder , E. Gronroos , P. Martinez , N. Matthews , A. Stewart , P. Tarpey , I. Varela , B. Phillimore , S. Begum , N. Q. McDonald , A. Butler , D. Jones , K. Raine , C. Latimer , C. R. Santos , M. Nohadani , A. C. Eklund , B. Spencer‐Dene , G. Clark , L. Pickering , G. Stamp , M. Gore , Z. Szallasi , J. Downward , P. A. Futreal , C. Swanton , N. Engl. J. Med. 2012, 366, 883. 22397650 10.1056/NEJMoa1113205 PMC4878653

[30] M. R. Stratton , P. J. Campbell , P. A. Futreal , Nature 2009, 458, 719. 19360079 10.1038/nature07943 PMC2821689

[31] D. Basanta , J. G. Scott , M. N. Fishman , G. Ayala , S. W. Hayward , A. R. Anderson , Br. J. Cancer 2012, 106, 174. 22134510 10.1038/bjc.2011.517 PMC3251863

[32] D. Dingli , F. A. d. C. C. Chalub , F. C. Santos , S. V. Segbroeck , J. M. Pacheco , Br. J. Cancer 2009, 101, 1130. 19724279 10.1038/sj.bjc.6605288 PMC2768082

[33] L. You , J. S. Brown , F. Thuijsman , J. J. Cunningham , R. A. Gatenby , J. Zhang , K. Staňková , J. Theor. Biol. 2009, 435, 78. 10.1016/j.jtbi.2017.08.022 28870617

[34] M. Egeblad , E. S. Nakasone , Z. Werb , Dev. Cell 2010, 18, 884. 20627072 10.1016/j.devcel.2010.05.012 PMC2905377

[35] E. C. de Bruin , T. B. Taylor , C. Swanton , Genome Med. 2013, 5, 101. 24267946 10.1186/gm505 PMC3978608

[36] D. F. Quail , J. A. Joyce , Nat. Med. 2013, 19, 1423. 24202395 10.1038/nm.3394 PMC3954707

[37] B. Crespi , K. Summers , Trends in ecology & evolution 2005, 20, 545. 16701433 10.1016/j.tree.2005.07.007

[38] A. R. Anderson , M. Hassanein , K. M. Branch , J. Lu , N. A. Lobdell , J. Maier , D. Basanta , et al , Cancer Res. 2009, 69, 8797. 19887618 10.1158/0008-5472.CAN-09-0437 PMC2783510

[39] R. Levins , Evolution in Changing Environments: Some Theoretical Explorations (2nd ed.), Princeton University Press, 1968.

[40] T. Nagylaki , Heredity 1975, 35, 67. 1058845 10.1038/hdy.1975.67

[41] S. Rosenblat , Journal of Mathematical Biology 1980, 9, 23.

[42] R. Levins , The American Naturalist 1979, 114, 765.

[43] P. L. Chesson , R. R. Warner , The American Naturalist 1981, 117, 923.

[44] P. L. Chesson , Journal of Mathematical Biology 1982, 15, 1.

[45] J. M. Cushing , Journal of Mathematical biology 1986, 24, 381. 3805902 10.1007/BF01236888

[46] A. L. Koch , J. Theor. Biol. 1974, 44, 373. 4829242 10.1016/0022-5193(74)90168-4

[47] T. Namba , J. Theor. Biol. 1984, 111, 369.

[48] T. Namba , S. Takahashi , Theoretical Population Biology 1993, 44, 374.

[49] P. Chesson , N. Huntly , The American Naturalist 1997, 150, 519. 10.1086/286080 18811299

[50] S. Tǎnase‐Nicola , I. Nemenman , J. R. Soc., Interface 2011, 9, 1354. 22112653 10.1098/rsif.2011.0695 PMC3350737

[51] A. E. Allahverdyan , S. G. Babajanyan , C.‐K. Hu , Phys. Rev. E 2019, 100, 032401. 31639934 10.1103/PhysRevE.100.032401

[52] A. E. Allahverdyan , C.‐K. Hu , Phys. Rev. Lett. 2009, 102, 058102. 19257560 10.1103/PhysRevLett.102.058102

[53] Z.‐X. Tan , J. M. Koh , E. V. Koonin , K. H. Cheong , Adv. Sci. 2019, 1901559. 10.1002/advs.201901559 PMC7001654 32042555

[54] K. H. Cheong , J. M. Koh , M. C. Jones , BioEssays 2019, 41, 1900027.

[55] J. Zhang , J. J. Cunningham , J. S. Brown , R. A. Gatenby , Nat. Commun. 2017, 8, 1816. 29180633 10.1038/s41467-017-01968-5 PMC5703947

[56] Y. M. Svirizhev , D. O. Logofet , Stability of Biological Communities, Nauka, Moscow 1978.

[57] J. Hofbauer , K. Sigmund , Evolutionary Games and Population Dynamics, Cambridge university press, 1998.

[58] M. Hussain , C. M. Tangen , D. L. Berry , C. S. Higano , E. D. Crawford , G. Liu , G. Wilding , et al , Cancer Res. 2013, 368, 1314. 10.1056/NEJMoa1212299 PMC3682658 23550669

[59] P. L. Kapitza , Zh. Eksp. Teor. Fiz. 1951, 21, 1354.

[60] A. Ridinger , N. Davidson , Phys. Rev. A 2007, 76, 013421.

