# Supplementary material for: Can Environmental Manipulation Help Suppress Cancer? Non‐Linear Competition Among Tumor Cells in Periodically Changing Conditions
Source: Adv Sci (Weinh). 2020 Jul 1;7(16):2000340. doi: 10.1002/advs.202000340 (PMC7435241; doi:10.1002/advs.202000340)
Supplement: Supplementary file 1 — Supporting Information [file ADVS-7-2000340-s001.pdf]

Supplementary Information

Derivation of Equation (8) and (9)

From (1) and (2), we have

$$\frac{dp_i}{dt} = p_i(r_i - \sum_k r_k p_k + N(\sum_{j,k} a_{kj} p_k p_j - \sum_k a_{ik} p_k)) \tag{13}$$

$$\equiv G_i(a, r, p, N)$$

$$\frac{dN}{dt} = N(\sum_k r_k p_k - N \sum_{j,k} a_{kj} p_k p_j) \tag{14}$$

For competing coefficients a_{kj}, we impose the condition $a_{ij}(\tau) = \bar{a}_{ij} + \widetilde{a}_{ij}(\tau)$. The reproduction coefficients r_i do not depend on time. The population densities are represented by Eqn (6). We assume that the oscillating parts of population densities are smaller than the slow-varying parts. Substituting (6) into (13) and expanding the right hand-side of (13) over ϵ_i we obtain the following:

$$\dot{\bar{p}}_i + \dot{\bar{p}}_\alpha \partial_\alpha \epsilon_i + \dot{N} \partial_N \epsilon_i + \omega \partial_\tau \epsilon_i = \tag{15}$$

$$(1 + \epsilon_\alpha \partial_\alpha) G_i(a(\tau), r, N, \overline{p}),$$

where, by Greek letters it is assumed the summation $A_\alpha B_\alpha = \sum_{\alpha=1}^n A_\alpha B_\alpha$, and $\partial_\alpha \equiv \frac{\partial}{\partial \overline{p}_\alpha}$. The total population size also depends on the oscillating part as seen from (14).

The fast variations ϵ_i are searched via expanding over $\frac{1}{\omega}$

$$\epsilon_i = \frac{1}{\omega}\epsilon_{i,1} + \frac{1}{\omega^2}\epsilon_{i,2} + ... \tag{16}$$

Substituting (16) into (15) and (14), we obtain

$$\dot{\bar{p}}_i + \frac{1}{\omega}\dot{\bar{p}}_\alpha \partial_\alpha \epsilon_{i,1} + \frac{1}{\omega}\dot{N}\partial_N \epsilon_{i,1} + \partial_\tau \epsilon_{i,1} + \frac{1}{\omega}\partial_\tau \epsilon_{i,2} = \tag{17}$$

$$= G_i(\overline{a}, r, N, \bar{p}) + G_i(\widetilde{a}, N, \bar{p}) +$$

$$+ \frac{1}{\omega}\epsilon_{\alpha,1}\partial_\alpha G_i(\overline{a}, r, N, \bar{p}) + \frac{1}{\omega}\epsilon_{\alpha,1}\partial_\alpha G_i(\widetilde{a}, N, \bar{p}) + O(\frac{1}{\omega^2}),$$

$$\dot{N} = N(\sum_k r_k(\bar{p}_k + \frac{1}{\omega}\epsilon_{k,1} + ...) - \tag{18}$$

$$- N\sum_{j,k}(\overline{a}_{kj} + \widetilde{a}_{kj})(\bar{p}_k + \frac{1}{\omega}\epsilon_{k,1} + ..)(\bar{p}_j + \frac{1}{\omega}\epsilon_{j,1} + ..)).$$

The terms in Eq. (17) have different timescales and amplitudes. First, we take the terms which are of order $O(1)$ and vary in the fast times:

$$\partial_\tau \epsilon_{i,1} = G_i(\widetilde{a}, N, \bar{p}) + O(\frac{1}{\omega^2}). \tag{19}$$

This equation can be integrated directly, since $\bar{p}_i(t)$ varies on the slow time. Taking into account Eq. (16) for the fast oscillating part, we obtain:

$$\epsilon_i = \frac{1}{\omega}G_i(\hat{a}, N, \bar{p}) + O(\frac{1}{\omega^2}), \tag{20}$$

where $\widehat{a}_i$ is the primitive of $\widetilde{a}_i$, $\partial_\tau \widehat{a}_i = \widetilde{a}_i$.

The dynamics of slow-varying part $\overline{p}$ is found from Eq. (17) after averaging the remained terms by fast time τ.

Using conditions (4) and (7), we have:

$$\dot{\bar{p}}_i = G_i(\overline{a}, r, N, \bar{p}) + \frac{1}{\omega}\overline{G_\alpha(\hat{a}, N, \bar{p})\partial_\alpha G_i(\widetilde{a}, N, \bar{p})} \tag{21}$$

$$\dot{N} = N(\sum_k r_k \bar{p}_k - N(\sum_{jk}\bar{p}_k \bar{p}_j \overline{a}_{kj} + \tag{22}$$

$$+\frac{1}{\omega}\overline{\sum_{jk}\bar{p}_k G_j(\hat{a}, N, \bar{p})\widetilde{a}_{kj}})).$$

In Eq. (22), we have used (20). The over lined quantities refer to averaged over fast time τ.

The slow-varying population densities and total population size dynamics (8,9) in the main text are derived using Eqs. (21) and (22) using the following:

$$\sum_{mn} \overline{\widetilde{a}_{in}\widetilde{a}_{im}}\bar{p}_m\bar{p}_n = \sum_{mnkl} \overline{\widetilde{a}_{kn}\widetilde{a}_{lm}}\bar{p}_m\bar{p}_n\bar{p}_k\bar{p}_l = 0 \tag{23}$$

This completes the derivation of (8) and (9).

Initial conditions

From the definition of slow- and fast-varying population densities, (Eq. (6) in the main text and Eq. (20), it follows that the initial condition for slow-varying densities differs from the initial condition of the overall (slow+fast) densities. Indeed, the initial condition for slow-varying part can be found from the following equation:

$$p(0) = \bar{p}(0) + \frac{1}{\omega} G_i(\hat{a}(0), N(0), \bar{p}(0)), \tag{24}$$

where it is assumed that the initial conditions of overall population densities and total population size are known. Thus, when the fast oscillations are activated, the dynamics of overall population densities and slow-varying densities appear to be converging after few oscillations. This effect is illustrated in the figures of the main text. This effect is important for some cases of the dynamics, *e.g* when there are more than one stable point and depending on the differences of the above quantities, the system may end up in different stable states. However, these differences are not crucial for the above reproduction and competition coefficients.
